# Dynamics and Surface Propensity of H^+^ and OH^–^ within Rigid Interfacial Water: Implications for Electrocatalysis

**DOI:** 10.1021/acs.jpclett.1c02493

**Published:** 2021-10-12

**Authors:** Rasmus Kronberg, Kari Laasonen

**Affiliations:** Research Group of Computational Chemistry, Department of Chemistry and Materials Science, Aalto University, P.O. Box 16100, FI-00076 Aalto, Finland

## Abstract

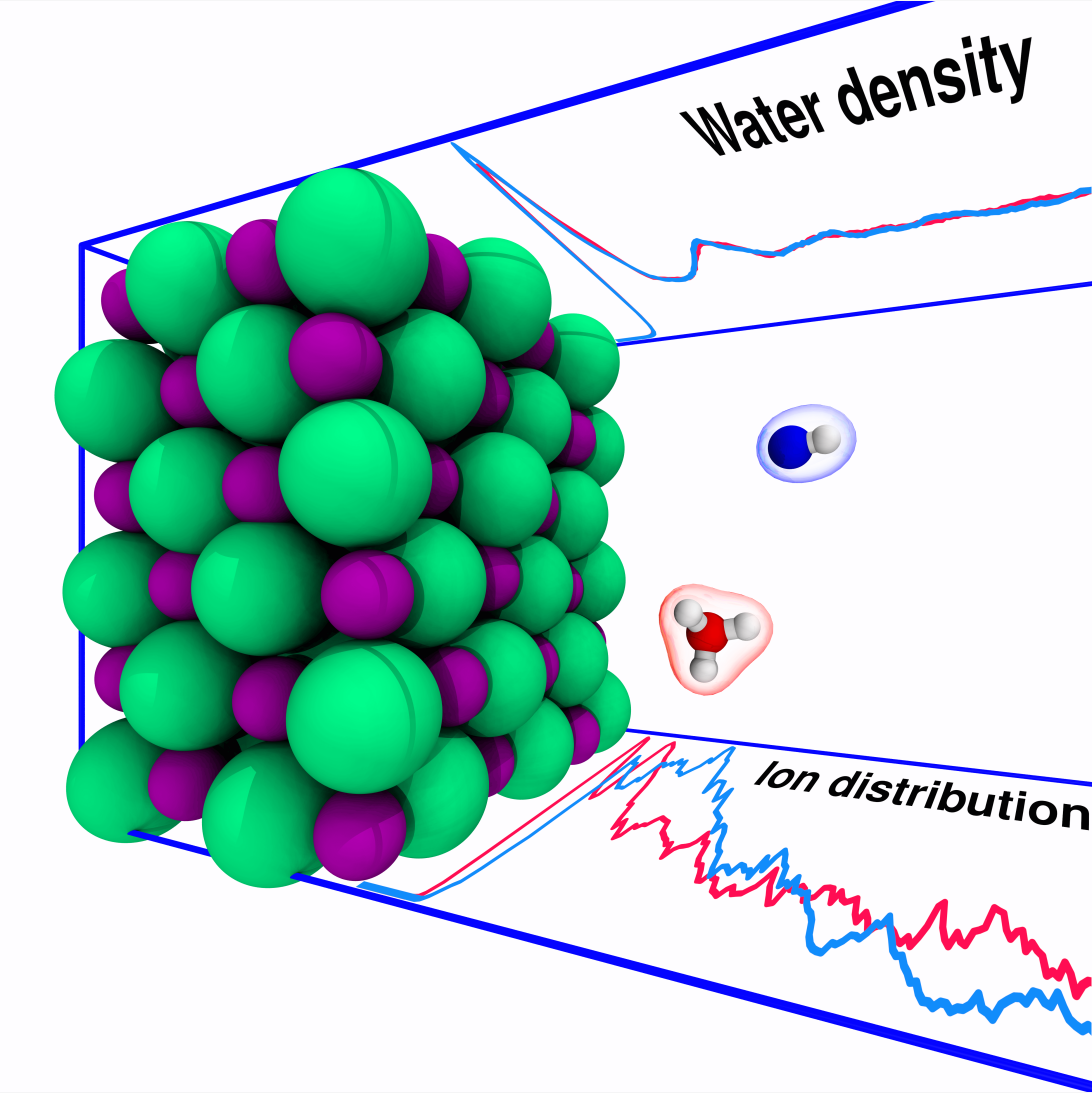

Facile solvent reorganization promoting ion transfer across the solid–liquid interface is considered a prerequisite for efficient electrocatalysis. We provide first-principles insight into this notion by examining water self-ion dynamics at a highly rigid NaCl(100)–water interface. Through extensive density functional theory molecular dynamics simulations, we demonstrate for both acidic and alkaline solutions that Grotthuss dynamics is not impeded by a rigid water structure. Conversely, decreased proton transfer barriers and a striking propensity of H_3_O^+^ and OH^–^ for stationary interfacial water are found. Differences in the ideal hydration structure of the ions, however, distinguish their behavior at the water contact layer. While hydronium can maintain its optimal solvation, the preferentially hypercoordinated hydroxide is repelled from the immediate vicinity of the surface due to interfacial coordination reduction. This has implications for alkaline hydrogen electrosorption in which the formation of undercoordinated OH^–^ at the surface is proposed to contribute to the observed sluggish kinetics.

Electrocatalyst development for renewable energy conversion technologies has benefited substantially from the establishment of computational methods as complementary tools for unraveling structure–activity relationships beyond the resolution limit of experiments.^1^ While considerable efforts have been directed toward disentangling the physical properties and catalytic activity of the solid electrode, the aqueous phase in contact with the electrocatalyst surface has received less attention. This is understandable considering the predominant role the electrode surface properties play in determining reaction rates, as well as the alleviated computational complexity of describing solid surfaces in a vacuum with implicit or *post hoc* consideration of solvent effects.^2^ Explicitly modeling the dynamic liquid phase is in contrast challenging owing to the large amount of statistical sampling required for appropriately converged structural and dynamical properties.

The influence of the liquid side should nevertheless not be ignored, but instead acknowledged as a crucial component in building a comprehensive picture of ubiquitous electrified interfaces. In addition to catalytic activation at the electrode, solvent dynamics may indeed significantly affect mass transport and contribute to electrochemical kinetics, such as recently proposed for the hydrogen evolution reaction (HER), i.e., the cathodic half-reaction of water electrolysis.^3^ In an attempt to explain the pH dependence of HER on Pt(111), the slow adsorption and evolution rates observed in alkaline media were ascribed to hindered water reorganization suppressing charge transfer through the electrochemical double layer (H_3_O^+^ in acid and OH^–^ in base). This is a consequence of the potential of zero free charge shifting to considerably positive voltages in alkaline solutions, creating a strong interfacial electric field due to the increased distance to the hydrogen adsorption potential. The interaction of water with this field makes the interfacial solvent rigid and difficult to reorganize.

Interfacial water reorganization as a descriptor of electrochemical rates is an intriguing model underscoring the importance of solvent dynamics in electrochemical kinetics unlike common adsorption energy (Δ*G*)-based heuristics.^4,5^ However, while intuitive and appealing, explicit atomistic support for the model is urgently required. In this Letter, we provide theoretical insight into this hypothesis through extensive (>0.5 ns) density functional theory molecular dynamics (DFTMD) simulations of acidic and alkaline NaCl(100)–water interfaces. The hydrophilic halite surface interacts strongly with water, thus providing an apt model of a highly rigid interfacial solvent structure. The electronic structure convergence of the wide-bandgap system is furthermore an order of magnitude faster than that of large metallic interfaces,^6^ allowing access to the unprecedented time scales necessary to appropriately sample the molecular dynamics of water self-ions, notably OH^–^. Subsequently, distance-dependent dynamics and surface propensities of hydronium and hydroxide are resolved, and we demonstrate that ion transfer to and within the interface is surprisingly facile despite the static water structure, thus challenging the proposed reorganization model. From a broader perspective, we note that the still poorly understood anisotropic dynamics and structural diffusion of water self-ions at interfaces and in confinement also have widespread implications within a range of other biological,^7−9^ geochemical,^10,11^ and technological^12−15^ processes besides electrocatalysis. This underscores the fundamental significance of the research topic presented here, which has received more attention in recent years.^16−19^

Structural and dynamical water properties are characterized to assert the rigidity of the interfacial solvent structure caused by the hydrophilic NaCl(100) surface. Results for both acidic and alkaline systems are included for the sake of completeness, although we expect minor differences between the two following averaging over all molecules among which the studied charge defects are merely local perturbations. While resolving the overall water structure is important to understand the environment in which the ions are embedded, a high degree of similarity of the results is also useful as an indicator of sufficient statistical convergence. Panels a and b of Figure 1 show illustrations of the model NaCl(100)–water interface and laterally averaged water density profiles across the investigated systems, respectively. The hydrophilic interaction causes pronounced density peaks 2.6 Å from the surface with maxima of 2.2–2.4 g cm^–3^. A second hydration layer emerges at 6.3 Å, after which the density gradually converges to the bulk value of 1.0 g cm^–3^. These density oscillations agree well with previous classical and first-principles simulations.^20,21^ On the basis of the observed hydration layer structure, we partition the solvent phase into eight 4.1 Å wide regions for subsequent distance-dependent analyses. Accounting for the mirror symmetry of the interfacial model, we obtain four distinct regions corresponding to the water contact layer (I), the diffuse region (IIa and IIb), and the bulk liquid (III).

**Figure 1:**
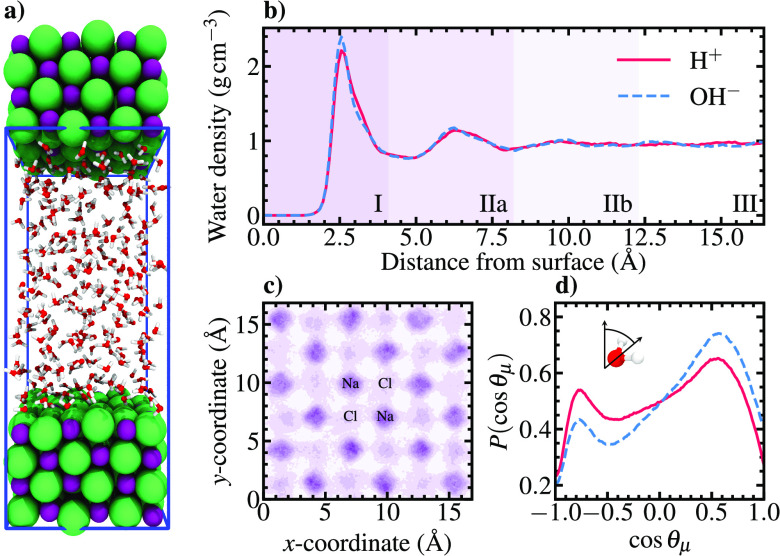
(a) Illustration of the simulated NaCl(100)–water interface. Purple and green spheres correspond to Na^+^ and Cl^–^ ions, respectively, while the connected red and white spheres represent explicit water molecules. The blue box marks the simulation cell boundaries. (b) Laterally averaged water density profiles of the studied acidic and alkaline systems. The solvent phase is partitioned into four 4.1 Å thick layers for subsequent distance-dependent analyses. Note that only half of the symmetric interfacial model is shown. (c) Spatial distribution of oxygen atoms within the water contact layer (I). Distributions of the acidic and alkaline simulations are superimposed using transparent-to-red and transparent-to-blue color maps so that dark purple regions correspond to high probability densities. (d) Dipole angle (cos θ_μ_) distribution of water molecules within layer I measured from the NaCl(100) surface normal. The legend in panel b applies also to panel d.

At both pH values, water molecules within layer I are preferentially pinned above Na^+^ ions as demonstrated by the surface-projected oxygen distributions (Figure 1c). Additionally, a slightly less pronounced population above Cl^–^ sites is evidenced. For reference, the water monomer adsorption energy is determined to be −0.53 eV (Figure S1), which can be compared with values of approximately −0.3 eV obtained for Pt(111).^22^ This underlines the significantly stronger halite–water interaction in contrast to the soft Pt(111)–water interface previously reported.^23^ Expectedly, the molecular orientations at the respective sites correspond to O-down and H-down water molecules with dipole angles of 55° and 140°, respectively, measured from the surface normal. The O-down peak is somewhat more pronounced in the alkaline system, which complies with the slightly increased density peak in Figure 1a.

The interfacial density, surface localization, and dipole angular distribution indicate a well-defined water contact layer irrespective of pH. To further characterize the water structure as a function of surface separation, distance-dependent hydrogen bond (HB) statistics are shown in Figure 2. While the mean HB count per bulk molecule is roughly 3.8 in accord with previous simulations,^20,24^ the interface substantially perturbs the tetrahedral HB structure in which one molecule ideally accepts and donates two HBs. Although the distribution of HBs in the insets of Figure 2 is rather similar within layers IIa–III, most water molecules at layer I have only three HBs. This number is further reduced for molecules pinned O-down at Na^+^ sites. The distance dependence of accepted and donated HBs shows that O-down water accepts on average one HB and donates ≤1.5 HBs, while adjacent H-down species accept two HBs and donate ≤1.5. The surface-induced disruption of the tetrahedral liquid structure and the enhanced packing of interfacial water reflect a pronounced occupation of interstitial sites, also confirmed by oxygen triplet angle and local structure index distributions (Figures S2 and S3).

**Figure 2:**
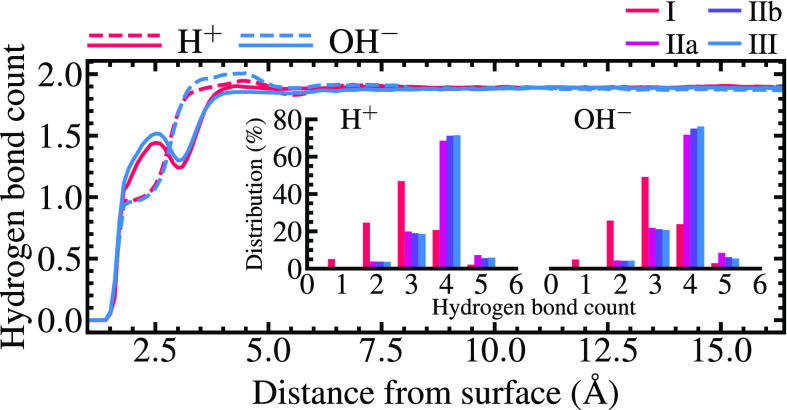
Average number of donated (solid lines) and accepted (dashed lines) hydrogen bonds per water molecule in the investigated systems as a function of surface separation. Insets show the distribution of total HB counts. A hydrogen bond is defined for O_d_···O_a_ distances of <3.5 Å and O_d_···H···O_a_ angles of >135°.^25^ The top right legend refers to the insets.

In addition to structural changes, a decline in water dynamics emerges with a decrease in surface separation. For both acidic and alkaline interfaces, the residence time of water molecules within layers I–III follows a decreasing trend where water at the contact layer is roughly twice as stationary as the bulk liquid (Figure 3a,b). The average residence time  is obtained by integrating the survival probability *P*(τ, Ω) of water molecules in a volume Ω
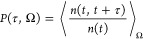
1where *n*(*t*) and *n*(*t*, *t* + τ) are the number of molecules at times *t* and every frame between *t* and *t* + τ continuously remaining within Ω, respectively.^26^ Importantly, the self-diffusion coefficient of a fluid in a heterogeneous system cannot be evaluated conventionally using Einstein or Green–Kubo relations. Instead, the parallel diffusion coefficients can be estimated following Liu et al.,^26^ who have shown that the mean-square displacement (MSD) of molecules within a confined volume parallel to a surface is proportional to the product of the diffusivity and the survival probability. Consequently, *D*_∥_(Ω) is obtained from eq 2
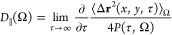
2i.e., the slope of MSD/4*P*(τ, Ω) at sufficiently long times where ballistic trajectories are excluded. When the data are plotted on a log–log scale (Figure 3c,d), the appropriately linear segments are identified as those having a slope of 1. These coincide with the time intervals of 4–7 ps for regions IIa–III and 7–15 ps for layer I. Long time lags are also excluded especially for the more dynamic regions where molecules leave the specified volume faster, resulting in poor averaging and deviation from linearity. The parallel diffusivities corroborate the conclusion reached on the basis of the survival probabilities; the water contact layer appears to be roughly twice as stationary as the bulk phase. Reassuringly, the bulk diffusion coefficient of ∼0.2 Å^2^ ps^–1^ agrees with the experimental^27^ value of 0.23 Å^2^ ps^–1^, indicating that the employed dispersion corrections and increased temperature indeed improve the PBE description of ambient liquid water (see Computational Methods). The obtained dynamical properties are also consistent with previous simulations.^28^

**Figure 3:**
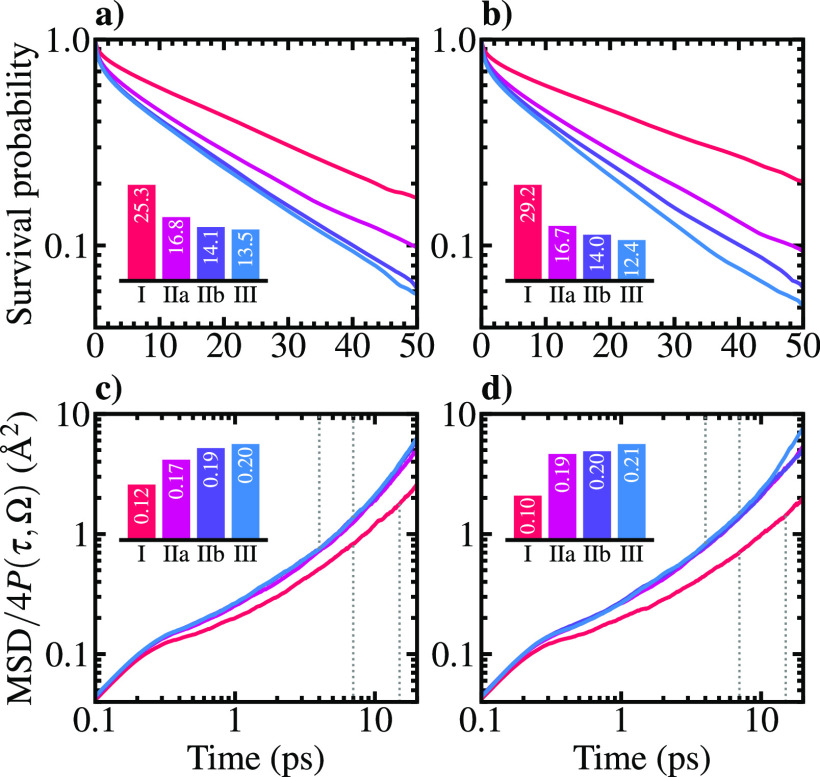
(a and b) Survival probability of water molecules within the defined solvent regions of the acidic and alkaline systems, respectively. Insets show the corresponding average residence times (ps) of the molecules obtained by integrating biexponential fits  to the survival probabilities. (c and d) MSD/4*P*(τ, Ω) of water molecules within the defined solvent regions of the acidic and alkaline systems, respectively. MSD refers to the mean-square displacement parallel to the NaCl surface. Insets show the corresponding parallel diffusion coefficients (Å^2^ ps^–1^) evaluated by eq 2 considering linear segments indicated by the dotted lines. The coloring follows the inset labels.

The main question to be assessed is whether the confirmed rigid interfacial solvent structure impedes the facile transport of water self-ions required for efficient electrocatalysis. Intriguingly, we observe that the declining water dynamics does not inhibit ion transfer from the bulk to the surface. The obtained ionic distributions shown in Figure 4a suggest in contrast pronounced surface concentrations. While the excess proton within layer I shows an equal preference for O-down and H-down water, the abundance of hydroxide in the immediate vicinity of the surface decays to zero. Instead, the majority of OH^–^ resides at the edge of layer I as well as within region IIa. Thus, while a clear surface propensity of both ions is evident, a difference in the distribution of the ions at the water contact layer is observed.

**Figure 4:**
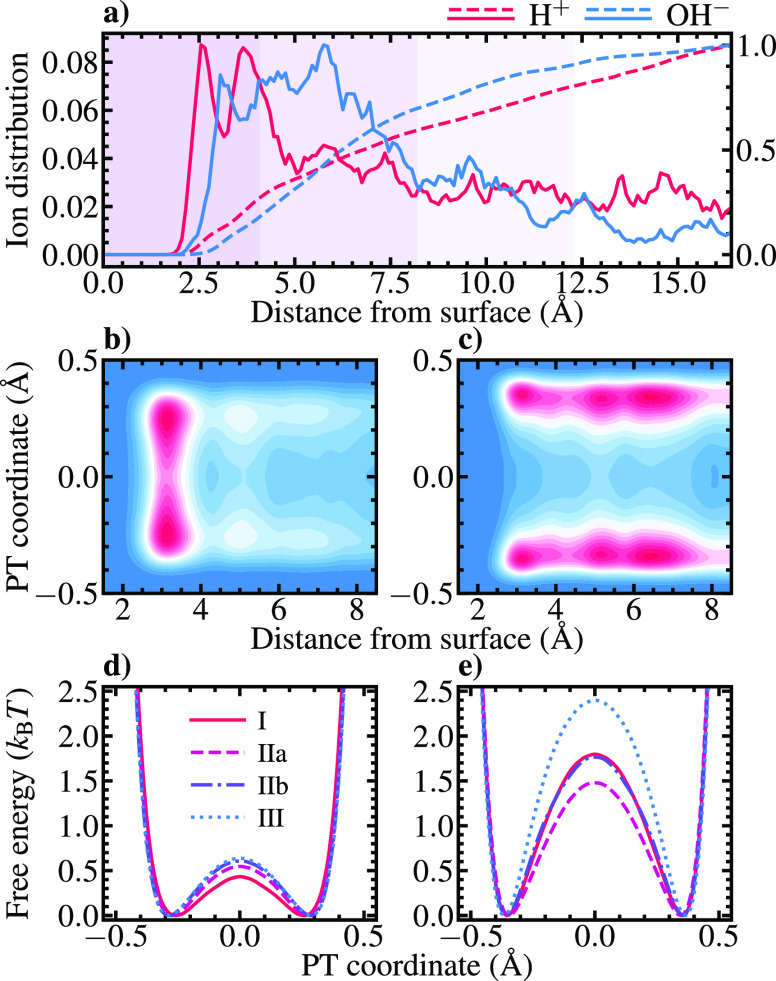
(a) Laterally averaged distribution of H_3_O^+^ and OH^–^ in the investigated acidic and alkaline systems (solid lines). The instantaneous positions of the ions along the DFTMD trajectories are determined as detailed in the Supporting Information. Dashed lines denote the respective cumulative distributions. (b and c) Distributions of the proton transfer coordinate as a function of surface separation for the acidic and alkaline systems, respectively. Red, white, and blue colors correspond to high, intermediate, and low probability densities, respectively. (d and e) Proton transfer free energy surfaces within the defined solvent regions of the acidic and alkaline systems, respectively. The legend in panel d applies also to panel e.

To comprehend the evidenced surface propensities, distance-dependent proton transfer (PT) dynamics are illustrated through the joint probability distribution of the proton transfer coordinate  and the surface separation of the water self-ions. Conventionally, the smallest value of δ is taken in each DFTMD frame to represent the position of the most active proton relative to the donor and acceptor oxygens (O_d_···H^+^···O_a_). As the present systems contain two charge defects (see Computational Methods), the two smallest δ are considered. The distributions in panels b and c of Figure 4 confirm the surface propensities of H_3_O^+^ and OH^–^, including the broader preferential distribution of OH^–^ extending through layer IIa. Expectedly, the population of small values of |δ| indicating high PT activity is pronounced for the acidic system, while in alkaline solution, the proton is more often localized on either oxygen.

As a direct assessment of the PT barriers, the PT coordinate distributions are re-evaluated for ions within the predefined solvent regions I–III. The Helmholtz free energy is given by
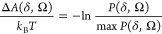
3where *P*(δ, Ω) is scaled by its maximum value so that the most probable δ yields a free energy of zero. The obtained results (Figure 4d,e) support the previous observations as PT barriers of both ions are largest in the bulk and decrease toward the interfacial region. While this decrease is monotonous for the hydronium transfer (0.6–0.4 *k*_B_*T*), the hydroxide ion showcases a minimum of roughly 1.5 *k*_B_*T* within layer IIa, in line with its increased probability density in this region. The barrier within layer I is in contrast virtually identical to that within layer IIb (1.8 *k*_B_*T*), while the corresponding value in the bulk is 2.4 *k*_B_*T*. Thus, the rigid solvent structure formed at the NaCl(100)–water interface does not impart Grotthuss kinetics of water self-ions. Transfer of the excess proton is in particular strongly promoted at the water contact layer, whereas hydroxide is most feasibly transported within the adjacent diffuse region IIa.

To explain the observed surface propensity and facilitated interfacial transport of H_3_O^+^ and OH^–^, we recall the established mechanisms of PT involving hydronium^29−31^ and hydroxide^31−33^ ions, respectively. For H_3_O^+^, PT is initiated by a coordination fluctuation between the first and second solvation shells of a hydronium that reduces the coordination of the acceptor oxygen. This thermally induced HB breakage (“presolvation”) allows the receiving oxygen to accept a HB from the hydronium along which PT subsequently occurs. Here, the hydrated proton is ideally thought to interchange between two Eigen H_9_O_4_^+^ structures via a Zundel H_5_O_2_^+^ complex with δ ≈ 0. Clearly, H_3_O^+^ transfer relies on HB breakage, and this is precisely what occurs at the interface where the bulk symmetry is disrupted. The decreased coordination of interfacial water facilitates surface-induced presolvation and explains the observed population enhancement and increased PT at the water contact layer. Additionally, as the ideal coordination of H_3_O^+^ is less than that of bulk water,^29^ the coordination decrease is not energetically unfavorable. In addition to the coordination number dependence in Figure 5a, the distribution of the hydronium oxygen triplet angle (Figure 5b) is practically independent of distance, confirming that H_3_O^+^ can maintain its ideal 3-fold hydration structure at the interface.

**Figure 5:**
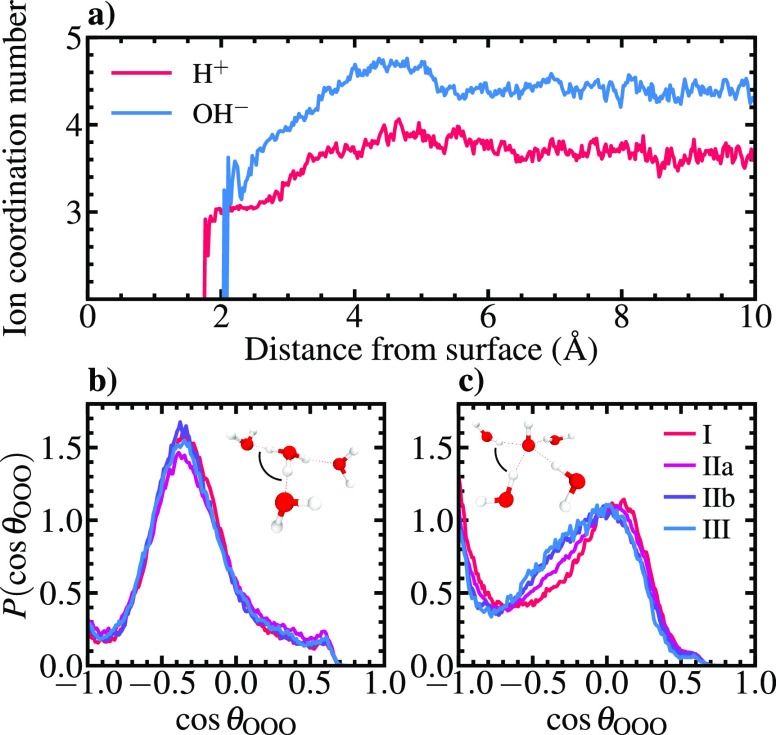
(a) Distance dependence of H_3_O^+^ and OH^–^ coordination numbers. (b and c) Distributions of water self-ion oxygen triplet angles (O···O···O) in the defined hydration layers of the acidic and alkaline systems, respectively. First solvation shell oxygens within 3.3 Å of the central H_3_O^+^/OH^–^ oxygen are considered.

This interpretation is analogous to the mechanism of facile PT in water-filled carbon nanotubes in which accelerated proton conduction is driven by favorable confinement-induced coordination reduction.^13^ Moreover, enhanced interfacial PT has been also observed at amorphous silica–water interfaces based on classical MD simulations employing a reactive all-atom potential.^18^ Despite the hydrophilic interaction inhibiting water diffusion, performed potential of mean force (PMF) calculations corroborate a PT barrier as low as 0.4 *k*_B_*T*, in reassuring agreement with our findings. By studying interfacial hydrogen bond autocorrelations, it is shown that continuous and intermittent HB lifetimes at the water contact layer are shorter and longer, respectively, than in the bulk, suggesting that PT is fast but rather localized to the surface, resulting in an enhanced interfacial hydronium population. Our extensive *ab initio* simulations support this conclusion and serve also as a rigorous validation of the employed reactive potential approach.

OH^–^ transport does not obey a mirror image mechanism compared to hydronium transport. While H_3_O^+^ is 3-fold coordinated in the Eigen form, panels a and c of Figure 5 demonstrate that OH^–^ is preferentially hypercoordinated, accepting four HBs in a square-planar conformation following delocalization of the lone pair electrons into a ring of negative charge around the oxygen.^32,33^ Additionally, roughly half of the ions donate a weak HB, resulting in an effective coordination number of ∼4.4. Hydroxide PT is initiated by a decrease in the coordination number of the OH^–^(H_2_O)_4_ species, and the defect is activated by formation of a transient H_3_O_2_^–^ complex with δ ≈ 0. Upon completion of PT, a new OH^–^(H_2_O)_3_ structure is formed, which subsequently accepts a fourth HB to attain its ideal hypercoordinated state. In stark contrast to the case of hydronium, the favorable hypercoordination explains why the hydroxide distribution rapidly decays to zero at the water contact layer with a concomitant increase in the PT barrier. While surface-induced perturbation of the HB network promotes H_3_O^+^ population and dynamics at layer I, formation of undercoordinated OH^–^ in the immediate vicinity of the surface through PT to the hydration layer above is infeasible. Conversely, the increased OH^–^ population and PT kinetics in region IIa are driven by enhanced hypercoordination (Figure 5a).

Parallels can be drawn between our findings and the behavior of protonic defects in nanoconfinement. Such simulations have been performed for water self-ions trapped between FeS mineral surfaces,^16,17^ where the spatial constraints strongly affect the structure and HB network of the confined water film. Regardless, hydronium has been shown to maintain its ideal hydration structure and fluxional nature surprisingly well, facilitating in-plane PT. This observation aligns well with our reasoning, notably that decreased coordination at the surface has less effect on hydronium solvation due to preferential undercoordination, also supported by the triplet angle distributions in Figure 5b. Conversely, increased confinement has been found to more significantly influence the interfacial dynamics of OH^–^.^17^ Indeed, the ideal square-planar hypercoordination demands a perpendicular orientation of the nascent hydroxide ion with respect to the surface to reach an appropriately stabilized resting state. This additional orientational constraint naturally decreases the probability of PT, which is supported by triplet angle distributions in Figure 5c showing enhanced structuring and planarity of hydroxide complexes at the water contact layer. In other words, formation of undercoordinated non-square-planar OH^–^ is clearly disfavored.

The propensity of H_3_O^+^ and OH^–^ for hydrophobic interfaces, e.g., air–water^34−38^ and graphene–water^19,39^ interfaces, has been debated widely owing to the importance of the phenomenon for interfacial acid–base chemistry. Most research efforts report that the hydronium displays a weak preference for the hydrophobic surface, while the community is significantly split concerning the hydroxide, likely due to widely different employed computational approaches ranging from empirical valence bond models and reactive force fields to first-principles simulations. A recent explanation^37^ for the repelling of OH^–^ from the immediate vicinity of the air–water interface relies on adverse changes in the solvation environment. Notably, surface-induced coordination reduction at the water contact layer is argued to decrease the number of energetically favorable ion–water interactions. This conclusion is equivalent to ours and suggests that the interfacial behavior of hydroxide is universal at any surface that breaks the bulk symmetry.

The interfacial propensity of OH^–^ has been also addressed by Grosjean et al.^19^ through constrained and unbiased DFTMD simulations of an alkaline graphene–water interface. Therein, rapid lateral ion transfer is argued to originate from facile proton shuttling between favorably oriented species. At the interface, an H-down hydroxide accepting a proton from an above-lying water molecule results in a flipped H-up species farther from the surface. The formed OH^–^ is unable to accept a proton from the above water molecules without reorientation, thus making PT back toward the surface kinetically more favorable than toward the bulk in a manner similar to what has been reported for the silica–water interface.^18^ Consequently, the preferred surface separation of OH^–^ is reported to range from 3 to 5 Å with an optimal physisorption distance of 3.3 Å, which appears to be sufficiently large for the hydroxide to maintain its ideal hypercoordinated square-pyramidal conformation. This view complies also with the results presented here considering the enhanced OH^–^ population and decreased PT barrier at 3–6 Å (Figure 4).

While recent electrochemical measurements^3^ emphasize the role of facile solvent reorganization and suggest that sluggish alkaline HER kinetics on Pt(111) is caused by suppressed OH^–^ transfer in rigid interfacial water, our results warrant a different explanation. Given the evidenced surface propensities and accelerated proton transfer kinetics, we argue that the decreased driving force is not an effect of a stationary water structure but is associated with the unfavorable formation of undercoordinated interfacial OH^–^ upon hydrogen electrosorption (H_2_O + e^–^ → H* + OH^–^). This proposition aligns with the presumed rate-limiting Volmer step of alkaline HER^40^ and also explains the improved performance of oxophilic electrocatalysts unlike platinum that can stabilize formed OH^–^ through chemisorption.^41^ This highlights the implications differences in the interfacial distribution, solvation, and transport of water self-ions may have for electrocatalysis.

On the basis of DFTMD simulations of unprecedented extent, we demonstrated that the migration of neither hydroxide nor hydronium is impeded by a rigid interfacial solvent structure. Although OH^–^ was observed to be repelled from the immediate vicinity of the hydrophilic surface, the ions display an overall surface propensity and decreased proton transfer barriers compared to the bulk solution. A theoretically well-founded explanation was proposed for the observations relying on the established mechanisms of proton transfer in acidic and alkaline media and the role of interfacial (pre)solvation. The disclosed findings are surprising and oppose an intuitive picture in which a rigid water structure and slow reorganization dynamics decrease surface concentrations by inhibiting ion transfer. This work considerably advances our general understanding of the nontrivial dynamics of H_3_O^+^ and OH^–^ in heterogeneous systems.

## Computational Methods

DFT calculations were performed within the hybrid Gaussian and plane waves framework^42^ as implemented in the CP2K/Quickstep^43^ code. The PBE^44^ exchange-correlation functional was applied together with dispersion corrections according to the DFT-D3(BJ) method.^45,46^ The valence orbitals of each element including the 2s and 2p semicore electrons of Na were expanded in molecularly optimized double-ζ plus polarization quality Gaussian basis sets.^47^ Ionic cores were described by norm-conserving GTH pseudopotentials.^48−50^ The orbital transformation method was used to solve the Kohn–Sham equations subject to an energy convergence criterion of 10^–6^ *E*_h_. The auxiliary plane wave basis was truncated with a 550 Ry kinetic energy cutoff. A similar value has been previously validated for NaCl dissolution simulations employing a nine-electron representation of sodium to circumvent the problem of nonlinear core correlation as done herein.^51^ We have retested this setup also for adsorption of the water monomer on NaCl(100) and found that it converges the adsorption energy to −0.53 eV in line with a reliable “gold standard” benchmark of −0.517 eV calculated at the CCSD(T) level of theory.^52^

A periodic four-layer *p*(3 × 3) NaCl(100) surface (Na_72_Cl_72_) was solvated by a roughly 3 nm thick water film containing 277 explicit H_2_O molecules. A NaCl lattice constant of 5.62 Å was optimized using the computational setup outlined above. The dimensions of the resulting simulation cell were 1.69 nm × 1.69 nm × 4.15 nm. To construct the simulated acidic and alkaline systems, two protons were either added to or removed from the bulk solvent region, yielding solution pH values of 0.4 and 13.6, respectively. The net charges of +2 and −2 carried by the systems were neutralized using a homogeneous background charge distribution. Born–Oppenheimer MD simulations were performed within the canonical (*NVT*) ensemble at a target temperature of 348.15 K maintained by a stochastic velocity rescaling thermostat.^53^ The applied temperature was chosen to reduce the extent of observed overstructuring of PBE water and to obtain the correct diffusive properties of water at room temperature.^25,54^ The increased temperature mimics also proton nuclear quantum effects known to affect the structural and dynamical properties of liquid water.^55,56^ Applying a time step of 0.5 fs, both systems were initially equilibrated for 20 ps, after which extensive production runs of >500 ps were performed. Trajectories were analyzed using the MDAnalysis^57^ Python package.

## Supporting Information Available

Lattice constant and water adsorption energy convergence, water oxygen triplet angular distribution, local structure index distribution, H^+^ and OH^–^ identification protocol, bulk water radial distribution, and water rotation dynamics (Figures S1–S5) (PDF)
