# Supplementary material for: Dynamics and Surface Propensity of H+ and OH– within Rigid Interfacial Water: Implications for Electrocatalysis
Source: J Phys Chem Lett. 2021 Oct 12;12(41):10128–34. doi: 10.1021/acs.jpclett.1c02493 (PMC8543677; doi:10.1021/acs.jpclett.1c02493)
Supplement: Supplementary file 1 — jz1c02493_si_001.pdf [file jz1c02493_si_001.pdf]

Supporting Information for:

Dynamics and Surface Propensity of H^+ and OH^- within Rigid Interfacial Water: Implications for Electrocatalysis

Rasmus Kronberg and Kari Laasonen*

Research Group of Computational Chemistry, Department of Chemistry and Materials Science, Aalto University, P.O. Box 16100, FI-00076 Aalto, Finland

E-mail: kari.laasonen@aalto.fi

Phone: +358 40 5570044

1 Lattice Constant and Water Adsorption

The NaCl lattice parameter was optimized using the computational setup outlined in the Computational Methods section in the main text (Figure S1a). The obtained equilibrium value of $a = 5.62\,\text{Å}$ is in fair agreement with the experimental reference value of 5.640 Å [1] as well as a high-level CCSD(T) estimate of 5.634 Å [2].

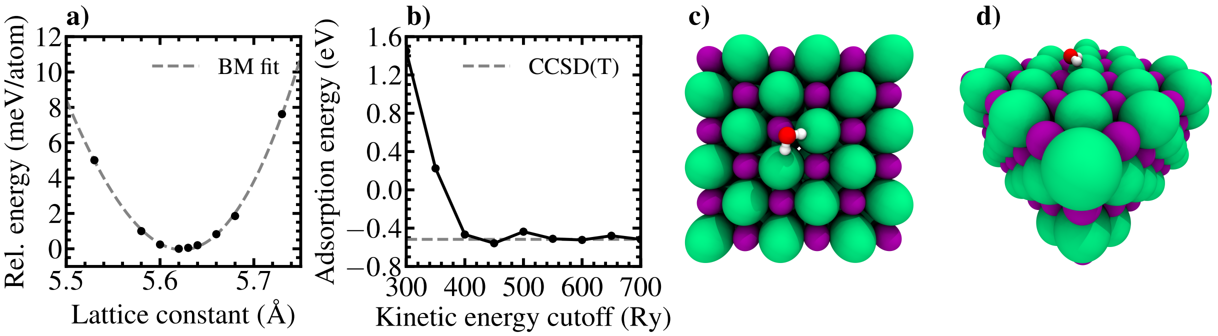

Figure S1: a) Relative energy per atom of a (3 × 3 × 3) bulk $Na_{108}Cl_{108}$ crystal as a function of the lattice constant. The Birch–Murnaghan (BM) equation of state has been fitted to the data. b) Adsorption energy of a water monomer on the NaCl surface slab as a function of the kinetic energy cutoff of the auxiliary plane wave basis. A high-level CCSD(T) result is marked by the dashed line for reference. c) Top and d) side views of the optimum adsorption configuration of a water monomer on NaCl.

Employing the optimized lattice constant, the kinetic energy cutoff of the auxiliary plane wave basis was optimized with respect to the water monomer adsorption energy (Figure S1b). Previously [3], a low cutoff of 280 Ry has been shown to suffice when describing Na using a one-electron pseudopotential. However, the partial overlap of the 3s valence orbital with the 2s and 2p orbitals introduces nonlinear core correlation, deeming either an explicit treatment of the semi-core electrons or electron density smoothing on the exchange–correlation grid necessary [4]. To avoid an *ad hoc* redefinition of the total energy using a smoothing operator we have opted to use a larger kinetic energy cutoff together with a more rigorous nine-electron representation of Na. A high cutoff of 550 Ry or larger is indeed found to be necessary for an appropriate description of the NaCl–water interaction, and consequently the structure and dynamics of the NaCl–water interface. Importantly, cutoff values of ≥ 550 Ry yield adsorption energies in reassuring agreement (max. rel. error 7 %) with previous computational studies [5,6], including an accurate CCSD(T) estimate of 0.517 eV. This correspondence applies also for the optimized structure of the adsorbed configuration (Figure S1c, d).

2 Water Oxygen Triplet Angular Distribution

To further assess the tetrahedral ordering in each defined hydration region, the oxygen triplet (O–O–O) angular distributions of water are analyzed. Expectedly, following considerable statistical sampling and averaging over all water molecules, the results for the acidic and alkaline interfaces in Figures S2a and S2b differ negligibly. In both systems bulk water is slightly disordered and characterized by a triplet distribution centered at 107°, which can be compared to the 109.5° angle of a perfectly tetrahedral structure. The increased packing of water molecules as a function of decreasing surface separation considerably broadens the distributions and shifts the peak positions gradually toward 104°. The surface-induced breakdown of the tetrahedral order is additionally indicated by the increasing amplitude of the shoulder around 50° to 60° associated with a highly distorted water structure with pronounced occupation of interstitial sites.

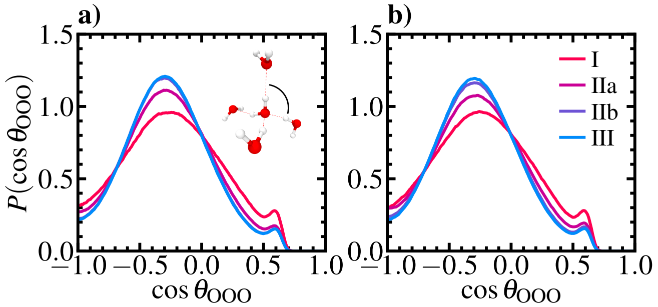

Figure S2: a,b) Distribution of oxygen triplet angles within the defined hydration layers of the acidic and alkaline systems, respectively. Atoms within a cutoff of 3.3 Å from the central oxygen are considered.

It is informative to compare the water oxygen triplet distributions to the ones of H_3O^+ and OH^- to assess differences in the hydration structures of the ions. Aside from a decreased average coordination number of ~ 3.6 (Figure 5a), the H_3O^+ angular distributions in Figure 5b reveals that the preferential hydration structure of the hydronium ion resembles rather closely that of bulk water. The triplet distribution peaks, however, at a stretched angle of roughly 112°, which coincides well with the ideal HOH angles of an isolated hydronium of ca. 113°[7]. Conversely, the angular distribution of the hydroxide complex (Figure 5c) is starkly distinct from the one of bulk water and that of hydronium. Notably, the preferred oxygen triplet angles are 90° and 180°, in line with the square planar/pyramidal hydration structure of OH^-.

3 Water Local Structure Index

The local structure index (LSI) is an order parameter that measures the extent of the gap between the first and second solvation shells of a water molecule[8]. It is therefore a sensitive probe of interstitial molecules in the ideally tetrahedral structure of water. Given a central water molecule, all oxygen–oxygen distances to its $n+1$ neighbors are calculated and ordered so that $r_1 < r_2 < \ldots < r_n < 3.7\,\text{Å} < r_{n+1}$. The LSI is then obtained as[9]

$$\text{LSI} = \frac{1}{n}\sum_{i=1}^{n}(\Delta_i - \langle\Delta\rangle)^2, \tag{S1}$$

where $\Delta_i = r_{i+1} - r_i$ and $\langle\Delta\rangle$ is the arithmetic mean of Δ_i. Consequently, low values of the LSI are found for "disordered" structures without a clear gap between first and second solvation shells, while high LSI values are found for ordered structures.

Resolving the LSI as a function of the separation from the NaCl surface yields the two-dimensional probability distribution presented in Figure S3. Clearly, high probability densities are observed for small LSI values at distances associated with the first and second hydration layers (layer I and region IIa). This corroborates the conclusion that the presence of the NaCl surface gradually reduces the HB network and that the strong interaction of water with the halite promotes increased packing of molecules, resulting in occupation of interstitial sites.

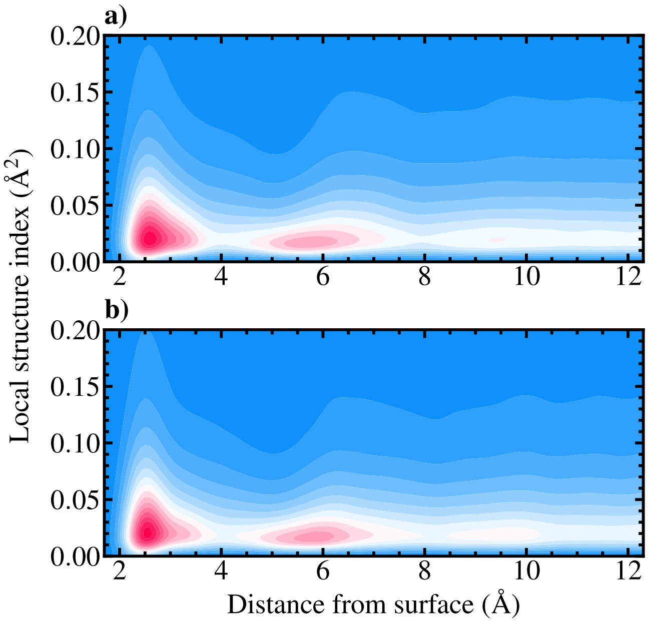

Figure S3: a, b) Local structure index as a function of surface separation for the acidic and alkaline systems, respectively. High–intermediate–low probability densities are indicated by the red–white–blue color scale.

4 H^+ and OH^- Identification Protocol

Special oxygens characterizing the position of H_3O^+ and OH^- were identified from the MD trajectories by analyzing the variance of r_{OH} distances to k-nearest neighbor (k-NN) hydrogens. Specifically, to locate an H_3O^+ entity, the distance from each oxygen to its 3 nearest neighboring hydrogens were calculated and the variance of the distances evaluated. The oxygen with the *smallest* variance in r_{OH} distances defines the instantaneous position of the hydronium (in our case two smallest variances as two excess protons were considered),

$$\mathrm{H_3O^+} \leftarrow \arg\min_{\mathrm{O}} \sigma^2 \left(|\mathbf{r}_\mathrm{O} - \mathbf{r}_{\mathrm{H(3\text{-}NN)}}|\right) \tag{S2}$$

Conversely, to locate an OH^- entity, the distance from each oxygen to its 2 nearest neighboring hydrogens were calculated and the variance of the distances evaluated. The oxygen with the *largest* variance in r_{OH} distances defines the instantaneous position of the hydroxide (in our case two largest variances as two protons were removed),

$$\mathrm{OH^-} \leftarrow \arg\max_{\mathrm{O}} \sigma^2 \left(|\mathbf{r}_\mathrm{O} - \mathbf{r}_{\mathrm{H(2\text{-}NN)}}|\right) \tag{S3}$$

5 Bulk Water Radial Distribution

To further confirm that water within layer III indeed inherits bulk-like properties, the OO and OH radial distribution functions (RDF) were computed and compared against a previous study [10] of explicitly bulk water (Figure S4).

Both OO and OH RDFs agree well with the reference results obtained using the same PBE-D3 functional as employed herein. A slight smoothing and decrease in peak heights is attributed to the higher simulation temperature of 348 K applied in the present work compared to the temperature of 324 K of the reference study. The integrated RDFs,

$$\mathrm{CN}_{ij}(r) = 4\pi n_j \int_0^r \mathrm{d}r' r'^2 g_{ij}(r'), \tag{S4}$$

where n_j is the number density of atomic kind j in the system, evaluated up to the first minima of 3.3 Å

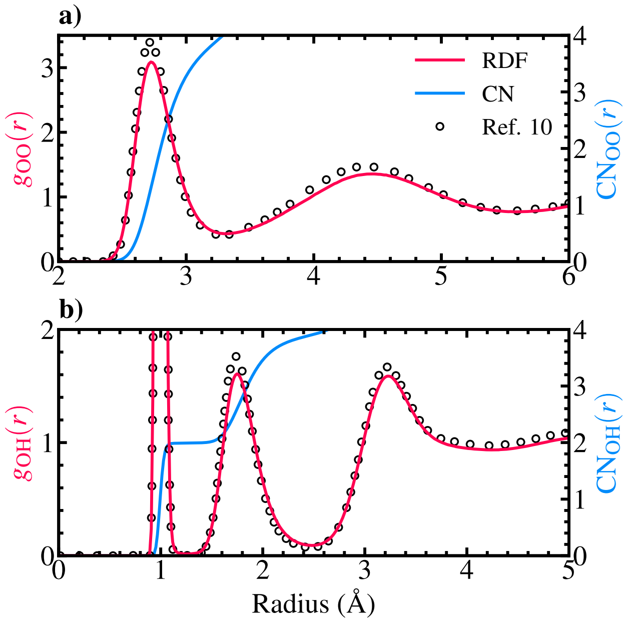

Figure S4: a) Oxygen–oxygen RDF (g_{OO}) of bulk water within layer III (left axis). The integrated RDF (cumulative coordination number CN_{OO}, right axis) is shown in blue. b) Oxygen–hydrogen RDF (g_{OH}) of bulk water within layer III (left axis). The integrated RDF (cumulative coordination number CN_{OH}, right axis) is shown in blue. Corresponding results from a study of PBE-D3/TZV2P bulk water is shown for reference[10].

(OO) and second minima of 2.5 Å (OH) yield coordination numbers of 4.0 and 3.9, respectively. We note that the simulations in the reference study were conducted using a larger doubly polarized TZV2P basis set compared to the DZVP basis employed here. As negligible differences are observed considering both RDFs (positions of peaks and minima, peak heights) and integrated coordination numbers, we conclude that the computationally more efficient DZVP basis adequately reproduces the properties of PBE-D3 bulk water, and that the water phase employed in the present study is of sufficient thickness to appropriately capture the full transition from interfacial to bulk water.

6 Water Rotation Dynamics

The dynamical properties of water can be rigorously characterized by studying the rotation dynamics and orientational relaxation of molecules. Given a water molecule with an initial orientation $\hat{\boldsymbol{\mu}}(t)$ specified by the unit dipole vector, the orientational autocorrelation at a later time $t + \tau$ is obtained by[10]

$$C(\tau) = \langle P_2[\hat{\boldsymbol{\mu}}(t) \cdot \hat{\boldsymbol{\mu}}(t + \tau)] \rangle, \tag{S5}$$

where $P_2(x) = (3x^2 - 1)/2$ is the second-order Legendre polynomial.

Computing the orientational autocorrelation function (ACF) for water molecules within the specified interfacial regions (I–III) yields the results presented in Figure S5. Surprisingly, the rotation dynamics are *accelerated* closer to the surface, although one would expect a slower water reorientation rate at the surface due to the asserted more rigid interfacial structure. This apparent anomaly is explained by considering the faster proton transfer dynamics (lower barriers) at the surface. Indeed, proton transfer events effectively change the dipole orientation of the water molecules exchanging a proton. This enables the apparent ultrafast rotation dynamics, which are almost twice as fast at the interface as compared to the bulk solution, in stark contrast to the overall water dynamics, which based on the diffusivities should be twice as *slow* at the interface. This observation is nonetheless in line with the increased surface population and proton transfer activity closer to the NaCl surface and demonstrates the surprising effect of protonic charge defects on conventional water rotation dynamics analyses.

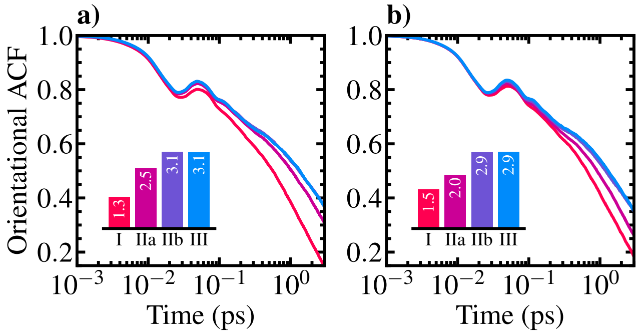

Figure S5: a, b) Orientational autocorrelation function for water molecules within the acidic and alkaline systems, respectively, and the specified interfacial regions. The results are plotted on a semi-logarithmic scale to better display the oscillation in the ACF at short time scales less than 100 fs. This oscillation is due to the libration of water molecules, which is mostly unaffected by the comparably slower proton transfer events (only the effect of fast proton transfer at the acidic water contact layer is slightly visible). The inset bar plots show the respective average relaxation times of the rotational motion, obtained through integration of biexponential fits ($a\mathrm{e}^{-\tau/\tau_1} + (1-a)\mathrm{e}^{-\tau/\tau_2}$) to the ACFs, $\bar{\tau} = \int_0^\infty \mathrm{d}\tau C(\tau, \Omega)$.

References

(1) Decker, D. L. High-pressure equation of state for NaCl, KCl, and CsCl. *J. Appl. Phys.* **1971**, *42*, 3239–3244.

(2) Doll, K.; Stoll, H. Cohesive properties of alkali halides. *Phys. Rev. B* **1997**, *56*, 10121.

(3) Holmberg, N.; Chen, J.-C.; Foster, A. S.; Laasonen, K. Dissolution of NaCl nanocrystals: an ab initio molecular dynamics study. *Phys. Chem. Chem. Phys.* **2014**, *16*, 17437–17446.

(4) VandeVondele, J.; Krack, M.; Mohamed, F.; Parrinello, M.; Chassaing, T.; Hutter, J. Quickstep: Fast and accurate density functional calculations using a mixed Gaussian and plane waves approach. *Comput. Phys. Commun.* **2005**, *167*, 103–128.

(5) Li, B.; Michaelides, A.; Scheffler, M. How strong is the bond between water and salt? *Surf. Sci.* **2008**, *602*, L135–L138.

(6) Kebede, G. G.; Spångberg, D.; Mitev, P. D.; Broqvist, P.; Hermansson, K. Comparing van der Waals DFT methods for water on NaCl(001) and MgO(001). *J. Chem. Phys.* **2017**, *146*, 064703.

(7) Tang, J.; Oka, T. Infrared spectroscopy of H_3O^+: The ν_1 fundamental band. *J. Mol. Spectrosc.* **1999**, *196*, 120–130.

(8) Duboué-Dijon, E.; Laage, D. Characterization of the local structure in liquid water by various order parameters. *J. Phys. Chem. B* **2015**, *119*, 8406–8418.

(9) Shiratani, E.; Sasai, M. Growth and collapse of structural patterns in the hydrogen bond network in liquid water. *J. Chem. Phys.* **1996**, *104*, 7671–7680.

(10) Bankura, A.; Karmakar, A.; Carnevale, V.; Chandra, A.; Klein, M. L. Structure, dynamics, and spectral diffusion of water from first-principles molecular dynamics. *J. Phys. Chem. C* **2014**, *118*, 29401–29411.
